# Analysis of the epidemiological characteristics between 2004 and 2017 and prediction of the changing pattern of other infectious diarrhea (OID) under COVID-19 in China

**DOI:** 10.1097/MD.0000000000031090

**Published:** 2022-10-21

**Authors:** Yujie Ge, Kai Wang, Jun Liu, Lingzhong Xu

**Affiliations:** a Centre for Health Management and Policy Research, School of Public Health, Cheeloo College of Medicine, Shandong University, Jinan, China; b Shandong University Center for Health Economics Experiment and Public Policy Research, Jinan, China; c NHC Key Laboratory of Health Economics and Policy Research, Cheeloo College of Medicine, Shandong University, Jinan, China; d Department of Research, The PLA Rocket Force Characteristic Medical Center, Beijing, China; e Affiliated Hospital of Jiangnan University, Wuxi, Jiangsu Province, China.

**Keywords:** ARIMA model, epidemiological characteristics, other infectious diarrhea, prediction

## Abstract

The study describes epidemiological features and transmission of other infectious diarrhea (OID) before and during the epidemic of COVID-19 in China, which lays a foundation for OID prevention and control. Incidence rate and mortality data of OID containing detailed epidemiological information such as date, age and region from 2004 to 2017, and total OID case number from 2018 to 2020 were obtained from the Data Center of China’s Public Health Science and the National Bureau of Statistics’ statistical yearbook. The Joinpoint regression model and *Z* test was used to analyze, while R language and ArcGIS 10.5 for drawing. The autoregressive integrated moving average model was used to predict the influence of COVID-19 on OID. The OID incidence rate increased from 31.69/10 million in 2004 to 92.42/10 million in 2017, and the mortality rate decreased from 1.82/10,000 to 0.14/10,000. The male to female incidence ratio was 1.39:1 (*P* < .001). The patients’ age showed a decreased trend with age *(P* < .001). The scattered children devoted the most OID incidence rate. The bimodal distribution of OID incidence was summer peak in northern China, 2 apparent peaks in central and eastern, and winter peak in southern. The autoregressive integrated moving average model predicted 1,406,557 in 2020, comparing the actual OID cases in 2020 to 1,062,277. Affected by the epidemic control measures of COVID-19, the number of OID cases declined by 32.4% (Absolute percentage error = 32.4%). The OID incidence rate in China continuously increased and showed a bimodal distribution in summer and winter with inconspicuous regional characteristics, gender and age susceptibility differences, and occupational differences. Meanwhile, COVID-19 significantly reduced OID incidence in 2020. The discoveries might bring a beneficial effect on OID prevention and control policies.

## 1. Introduction

Despite significant advances in sanitation and public health awareness, diarrheal disease remains 1 of the leading causes of the global burden of disease,^[1,2]^ the eighth leading cause of death worldwide, accounting for 16 million fatalities in 2016 alone.^[3]^ According to the law of the People’s Republic of China on the prevention and control of infectious diseases, other infectious diarrhea (OID) refers to infectious diarrhea other than cholera, dysentery, typhoid fever, and paratyphoid fever, which is a group of intestinal infectious diseases caused by viruses, bacteria, and protozoa, such as the common pathogens *rotavirus*, *Vibrio parahaemolyticus*, *Salmonella*, *enterotoxigenic Escherichia coli*, *Giardia lamblia*. This class C notifiable infectious disease is legally reported in China and has been included in the disease monitoring information reporting management system since 2004. The incidence rate of China’s OID diseases has always been the top of intestinal infectious diseases with a high incidence rate and low mortality rate,^[4]^ which has become an essential concern in preventive medicine and clinical medicine. The rates are not only indicators for measuring the level of health in a country but also an index reflecting the country’s social and economic level and the nation’s comprehensive quality.

The pandemic of COVID-19 has brought significant challenges to the world. Up to now, it involves more than 548 million confirmed cases and 6 million confirmed deaths in more than 200 countries and regions.^[5]^ China has implemented the most effective public health measures in the world to reduce the transmission of COVID-19, including containment and closure policies, strict closed-off management, wearing masks, promoting hand hygiene, environmental disinfection, physical distancing, and isolation.^[6]^ These non-pharmacological implements have effectively reduced the spread of COVID-19 and the risk of other infectious diseases’ transmission. Some research have reported the epidemiological features of other notifiable diseases in China, such as sexually transmitted diseases and other respiratory infectious diseases, that the factors included spatial and temporal characteristics and age group distribution changed.^[7,8]^ Sync with this, diarrheal cases have been found significantly reduced in England since COVID-19 control measures.^[9]^ However, the impact of China’s measures against COVID-19 on the transmission of OID was not mentioned.

Research has been done in China to investigate the epidemiological characteristics of OID; however, most of them just discussed the data of a specific district and the population distribution, especially the occupational distribution, is ignored. Therefore, we focus on OID, intending to describe its comprehensive epidemiological features and explore its transmission before and during the epidemic of COVID-19 in China, and then provide supplement the OID researched data and lay a foundation for the prevention and control of OID in China.

## 2. Materials

### 2.1. Data sources and data collection

The incidence and mortality data of OID were obtained from the Data Center of China’s Public Health Science (http://www.phsciencedata.cn/Share/ky_sjml.jsp), which is the primary data center of the National Population Health Science data sharing platform in China. The population data came from the National Bureau of Statistics’ statistical yearbook over the years. It should be noted that, because official statistics tend to be produced with long lags, the detailed data was from January 1, 2004, to December 31, 2017, which was used to describe the epidemiology characteristics of OID, while only incidence rate can be found in 2018 to 2020, which was added to build the OID autoregressive integrated moving average (ARIMA) model, verify and predict the OID incidence.

OID data was extracted, including incidence and mortality of 31 provinces and regions (autonomous regions and municipalities directly under the central government) in China. To assess the epidemiological trends and hotspots of OID in mainland China, the data on OID, including the number of cases and deaths, the incidence and mortality, were stratified by date (month and year), age and region.

We defined the incidence rate (100 thousand per person) as the number of cases per year divided by population size. The mortality rate is the number of deaths per year divided by the number of cases per year. This study is the secondary analysis of online reports data and other publicly available data and does not access any individually identifiable data. Therefore, ethical review is waived.

### 2.2. Statistical analysis

#### 2.2.1. Epidemiological description.

The descriptive epidemiological method was used to analyze the characteristics of OID. The Joinpoint regression model analyzed the data on infectious diarrhea, and *Z* test was used to evaluate whether there was a significant difference in the trend. Relevant statistical analysis contents were completed by SAS (version 9.4, SAS Institute Inc, Cary, NC), R language was used for drawing, and Joinpoint software (version 4.7, Statistical Research and Applications Branch, National Cancer Institute, USA) was used for trend analysis. Map drawing used ArcGIS 10.5 software (ESRI Inc., Redlands, CA).

#### 2.2.2. Prediction using ARIMA model.

We assumed that 2020 was a sensitive year for assessing COVID-19 epidemic policy to affect the spread of OID. We used the number of reported OID patients per year from 2004 to 2019 as the baseline, and used ARIMA model to complete the prediction of the number of OID in 2020. The establishment of ARIMA prediction model is completed in 4 steps: time series stationarity test, model identification, parameter estimation and model test and evaluation, and model fitting and application.

#### 2.2.3. Evaluation of the influence of COVID-19.

In order to evaluate the influence of COVID-19 control measures on the incidence of OID, we calculated the decline rate of the predicted number of patients in 2020 compared with the actual number of patients in 2020. According to Jia’s research, we introduce the concept of absolute percentage error (APE) to evaluate the influence of OID during COVID-19 epidemic. APE was calculated as the formula: APE = (Number _actual reported cases_−Number _predicted cases by ARIMA_)/Number _actual reported cases_.

## 3. Results

### 3.1. The epidemic of OID in mainland China from 2004 to 2017

**Figure 1.**
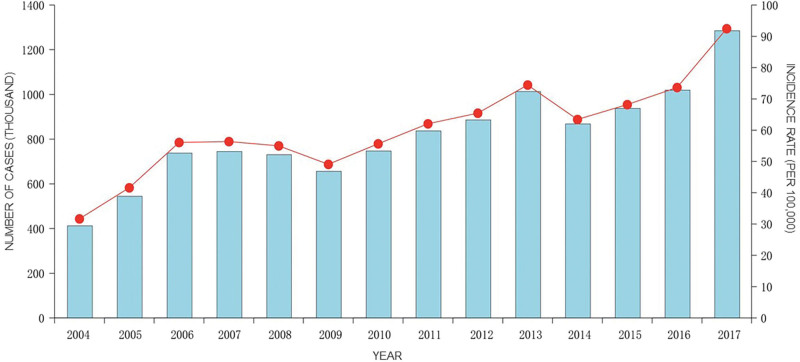
The annual incidence rate and number of OID cases reported in mainland China from 2004 to 2017. OID = other infectious diarrhea.

From 2004 to 2017, a total of 11,414,247 cases of OID were reported in 31 provinces and regions, involving 574 deaths (Table 1). The average annual incidence rate was 60.64 per 100,000, and the mortality rate was 5 per 100,000. During the study period, the incidence rate of OID increased from 31.69/10 million in 2004 to 92.42/10 million in 2017. The highest incidence rate of OID was observed in 2017 (92.42/10 million) (Fig. 1). The mortality rate decreased from 1.82 per 10,000 confirmed OID cases in 2004 to 0.14 per 10,000 in 2017.

**Table 1:** The average annual incidence of OID in mainland China from 2004 to 2017.

Provinces and regions	Number of cases	Number of deaths	Incidence rate (1/100,000)	Mortality rate (%)	AAPC (95% CI); *P* value
Anhui	776,078	12	90.81	0.0015	13.1 (11.3 to 14.9); *P* < .05
Beijing	672,875	24	251.35	0.0036	−3.9 (−7.4 to −0.3); *P* < .05
Fujian	288,764	21	55.58	0.0073	7.6 (4.7 to 10.6); *P* < .05
Gansu	127,861	9	35.52	0.0070	10.7 (8.6 to 12.9); *P* < .05
Guangdong	1,554,709	69	112.04	0.0044	4.4 (1.6 to 7.3); *P* < .05
Guangxi	540,031	107	81.04	0.0198	9.5 (3.6 to 15.7); *P* < .05
Guizhou	103,023	30	20.52	0.0291	7.3 (5.4 to 9.2); *P* < .05
Hainan	47,332	1	38.70	0.0021	4.6 (1.3 to 8.0); *P* < .05
Hebei	695,342	20	69.27	0.0029	6.6 (1.9 to 11.5); *P* < .05
Henan	547,022	23	41.32	0.0042	9.1 (7.2 to 11.0); *P* < .05
Heilongjiang	69,811	7	13.05	0.0100	1.5 (−1.5 to 4.6); *P* = .3
Hubei	337,350	8	41.78	0.0024	31.6 (9.3 to 58.6); *P* < .05
Hunan	302,910	24	32.85	0.0079	10.0 (7.2 to 12.9); *P* < .05
Jilin	19,806	1	5.17	0.0050	5.1 (−0.5 to 11.0); *P* = .1
Jiangsu	197,218	3	17.99	0.0015	2.7 (−2.2 to 7.9); *P* = .3
Jiangxi	366,055	44	58.63	0.0120	8.4 (3.6 to 13.4); *P* < .05
Liaoning	210,426	2	34.66	0.0010	14.4 (−0.0 to 31.0); *P* = .1
Nei Monggol	36,611	5	10.60	0.0137	24.1 (2.2 to 50.8); *P* < .05
Ningxia	109,170	1	122.65	0.0009	4.5 (0.5 to 8.7); *P* < .05
Qinghai	37,779	5	47.60	0.0132	12.5 (6.3 to 19.1); *P* < .05
Shandong	593,378	7	44.21	0.0012	13.4 (11.2 to 15.8); *P* < .05
Shanxi	186,099	11	37.67	0.0059	9.9 (0.5 to 20.1); *P* < .05
Shaanxi	286,584	23	54.66	0.0080	6.7 (4.4 to 9.0); *P* < .05
Shanghai	71,123	0	22.78	0.0000	2.6 (−3.4 to 9.0); *P* = .4
Sichuan	492,856	38	43.19	0.0077	−0.4 (−5.3 to 4.7); *P* = .9
Tianjin	531,725	0	289.25	0.0000	8.8 (−2.0 to 20.7); *P* = .1
Tibet	1500	0	3.53	0.0000	−9.6 (−28.0 to 13.5); *P* = .4
Xinjiang	217,932	25	70.76	0.0115	2.7 (−1.8 to 7.3); *P* = .2
Yunnan	162,354	13	25.14	0.0080	14.2 (10.7 to 17.9); *P* < .05
Zhejiang	1,485,164	21	198.52	0.0014	0.4 (−2.0 to 2.9); *P* = .7
Chongqing	345,359	20	84.72	0.0058	9.3 (7.5 to 11.2); *P* < .05
Total	11,414,247	574	60.64	0.005	5.38 (3.6 to 7.2); *P* < .05

OID = Other infectious diarrhea.

### 3.2. The population distribution of OID in mainland China from 2004 to 2017

**Figure 2.**
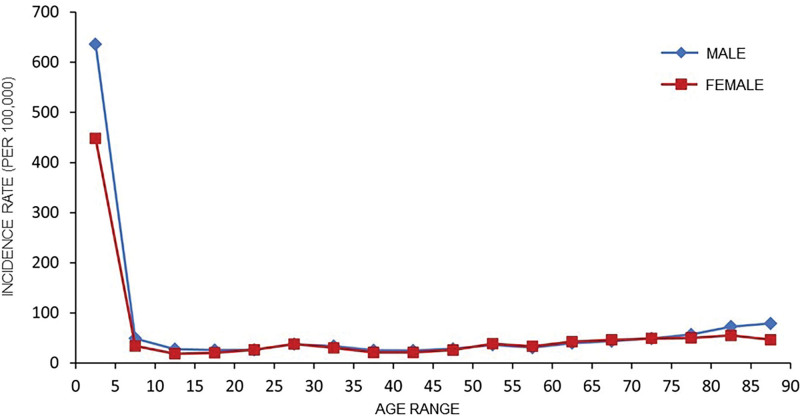
The gender and age distribution of OID in mainland China from 2004 to 2017. OID = other infectious diarrhea.

The gender and age distribution were shown in Figure 2. In mainland China from 2004 to 2017, 6,628,754 cases of OID were male (58.08%), and 4,785,253 cases were female (41.92%). The male to female OID incidence ratio was 1.39:1, and the difference was statistically significant (*P* < .001). The patients’ age showed a decreased trend with age *(P* < .001). The peak incidence rate was 0 to 4 years old group (550 per 10 million), accounting for 51% of the total cases (Fig. 2).

From the perspective of occupational distribution, the most OID incidence rate was among scattered children, with 5,684,301 cases (49.80%), followed by farmers 2,005,615 cases (17.57%), students 765,601 cases (6.71%), housework and unemployment group 540,905 cases (4.74%), and workers 507,012 cases (4.44%) (Table 2).

**Table 2:** The occupational distribution of OID in mainland China from 2004 to 2017.

Occupation	Number of cases	Constituent ratio (%)
Scattered children	5,684,301	49.80
Farmer	2,005,615	17.57
Student	765,601	6.71
Housework and unemployment group	540,905	4.74
Worker	507,012	4.44
Retirees	475,544	4.17
Childcare children	341,813	2.99
The cadre staff	313,186	2.74
Commercial service	141,810	1.24
Laborer	122,107	1.07
Teacher	76,374	0.67
Medical staff	37,201	0.33
Restaurant servants	28,192	0.25
Civil servants	23,467	0.21
Public servants	8321	0.07
Commercial personnel	6265	0.05
Herdsman	6256	0.05
Fisher	5582	0.05
Seafarers and long-Distance drivers	2947	0.03
Childminders and nannies	1778	0.02
Other	200,863	1.76
unknown	119,107	1.04

OID = Other infectious diarrhea.

### 3.3. The temporal distribution of OID in mainland China from 2004 to 2017

**Figure 3.**
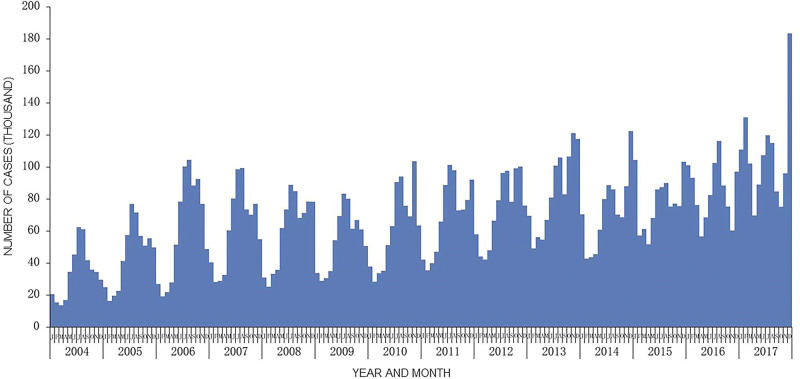
The temporal distribution of OID in mainland China from 2004 to 2017. The radar chart is clockwise for 12 months, and its radius shows the OID incidence rate (1/100,000). OID = other infectious diarrhea.

**Figure 4.**
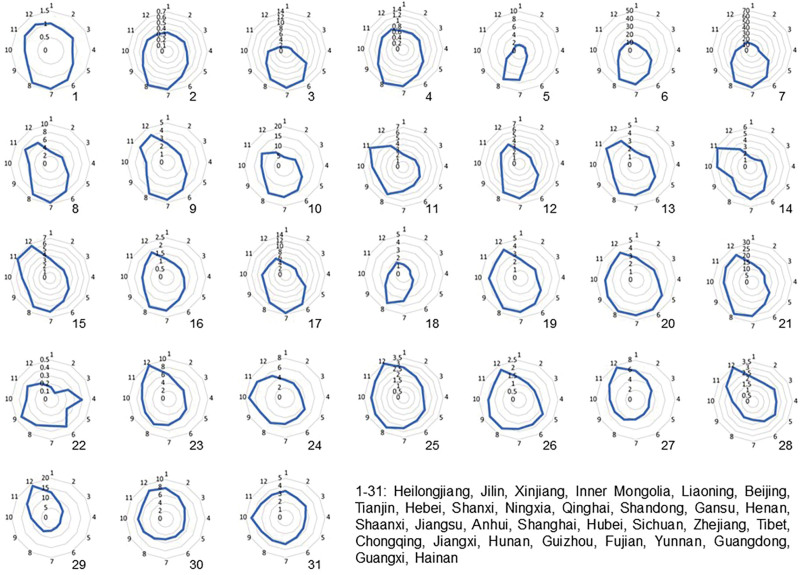
The number of OID cases per month reported in mainland China from 2004 to 2017. OID = other infectious diarrhea.

Radar charts were used to present the seasonal characteristics of OID in 31 provinces and regions in mainland China from 2004 to 2017 (Fig. 3). The seasonal trend of OID incidence peaks over the years was basically the same in China, with a bimodal distribution, which showed the summer peak occurred from July to August and the winter peak appeared from November to December (Fig. 4). It was found that OID increased significantly in summer (July to August) in northern China; meanwhile, there were 2 apparent peaks in central and eastern China, and obviously, winter peaks occurred in southern China (Guangdong Province and Guangxi Zhuang Autonomous Region).

### 3.4. The geographical distribution of OID in mainland China from 2004 to 2017

**Figure 5.**
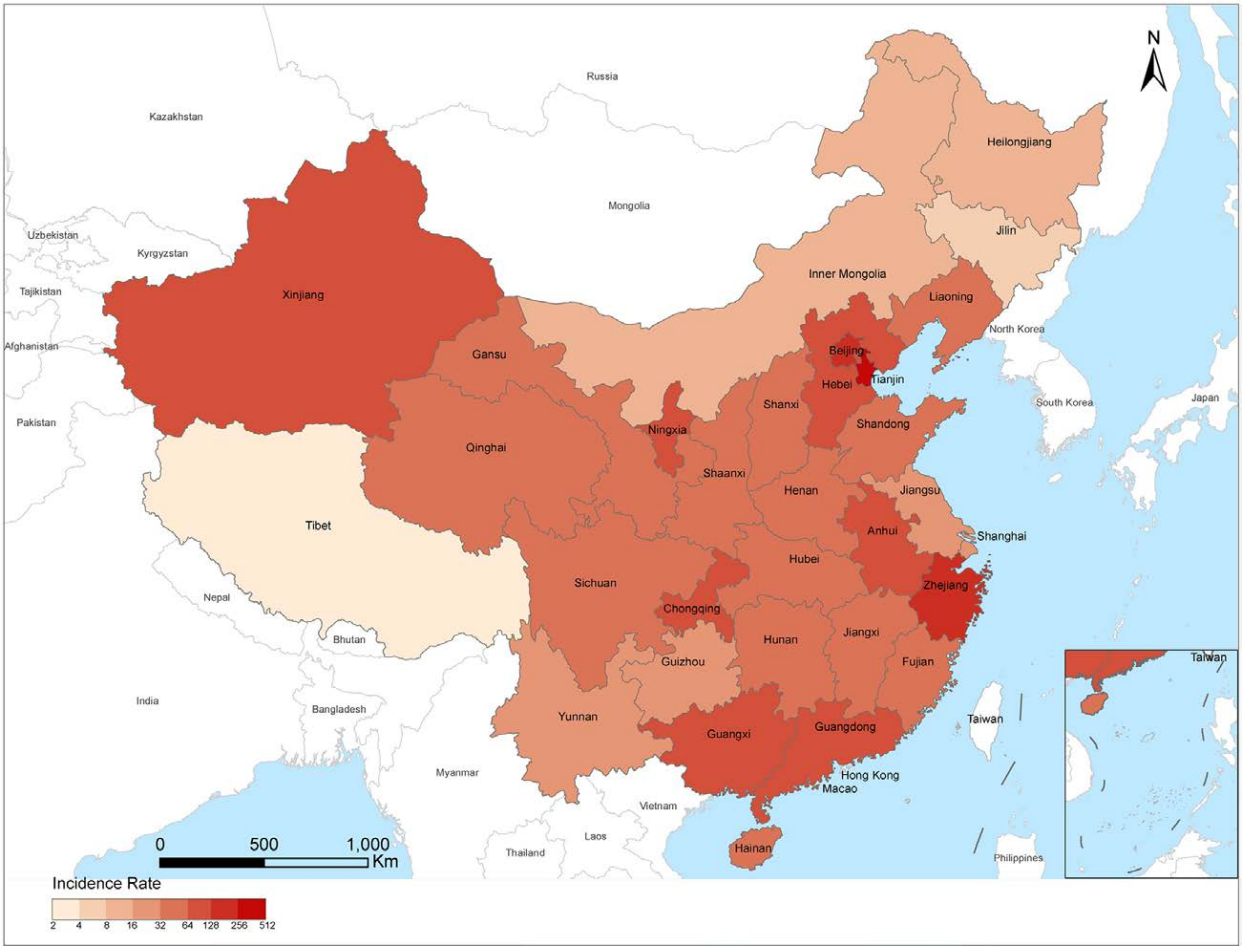
The geographical distribution of OID in mainland China. OID = Other infectious diarrhea.

From 2004 to 2017, the top 5 regions with the largest increases in incidence were Hubei Province with 31.6% (95% Confidence interval (CI): 9.3 to 58.6; *P* < .05), Inner Mongolia Autonomous Region with 24.1% (95% CI: 2.2 to 50.8; *P* < .05), Yunnan Province with 14.2% (95% CI: 10.7 to 17.9; *P* < .05), Anhui Province with 13.1% (95% CI: 11.3 to 14.9; *P* < .05) and Gansu Province with 10.7% (95% CI: 8.6 to 12.9; *P* < .05). The incidence rate was reduced 3.9% in Beijing (95% CI: -7.4 to −0.3; *P* < .05) during 2004-2017. As shown in the statistical map, Tianjin (289.25/10 million), Beijing (251.35/10 million), Zhejiang (198.52/10 million), the Ningxia Hui Autonomous Region (122.65/10 million) and Guangdong (112.04/10 million) expressed higher incidence (Table 1). The regional distribution of incidence rate is shown in Figure 5.

### 3.5. The predicted incidence of OID in mainland China in 2020

**Figure 6.**
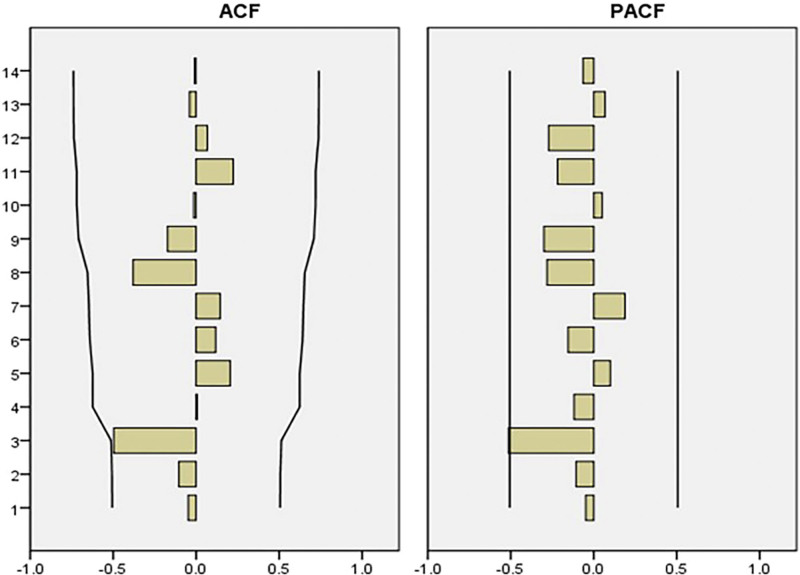
ACF and PACF diagrams of residuals in ARIMA model. ACF = autocorrelation function, ARIMA = autoregressive integrated moving average, PACF = partial autocorrelation function.

**Figure 7.**
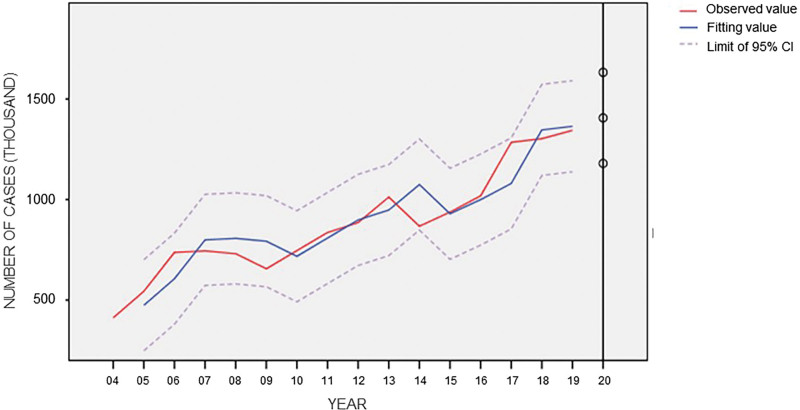
ARIMA model fitting and prediction of the newly reported case number of OID. ARIMA = autoregressive integrated moving average, OID = other infectious diarrhea.

The annual incidence of OID from 2004 to 2018 was used for model fitting, and the model was identified and preliminarily judged. When ARIMA (0, 1, 0) was constructed, the analysis results showed that Autocorrelation Function showed tailing attenuation and Partial Autocorrelation Function showed 1-step truncation, which could judge that the incidence rate data was stable. The residual sequence is tested and shown as a white noise sequence, which can be considered as no identifiable information in the residual error. The number of cases predicted by this model in 2019 is compared with the actual number of cases in 2019. The actual number of cases in 2019 is within the 95% CI range of the predicted value, and the relative error is 1.54% (Fig. 6). Finally, the ARIMA prediction model predicted the number of OID patients in 2020 to be 1,406,557 and the actual number of OID patients in 2020 to be 1,062,277. APE = 32.4% (Fig. 7).

## 4. Discussion

The present study found that from 2004 to 2017, the incidence rate of OID in China gradually increased from 31.69/100,000 to 92.42/100,000. The revelation of dynamic change characteristics is of great significance for future epidemiological prevention and control strategies. It should be noted that this does not mean that the situation of infectious diseases prevention and control in China is severe. On the contrary, China’s overall prevention and control of notifiable infectious diseases has achieved good results, with the incidence of 21 infectious diseases and the mortality rate of 17 infectious diseases showing a downward trend, which is inseparable from a series of infectious disease prevention and control strategies formulated by the state. Actually, the revelation of dynamic change means that the epidemic trend of OID contradicts the effectiveness of infectious disease prevention and control in the same period, which is the interest of epidemiological research.

Generally, the reported OID incidence rate increase should be attributed to the following factors. First, the expansion of the pathogenic spectrum, especially viral pathogens, has promoted the prevalence of OID and caused increasing outbreaks and public health emergencies which usually bring a large number of cases.^[10]^ A pathogenic study suggested that norovirus outbreaks were the leading cause of OID diseases after 2013.^[10]^ Second, With the continuous improvement of pathogen detection ability, especially the rapid PCR diagnosis technology has been widely used in hospitals at all levels in China, and the diagnostic sensitivity and specificity of OID have been greatly enhanced.^[11,12]^ The improvement in the level of infection diagnosis has led to an increase in the number of reported cases. Third, the network reporting system of infectious diseases (in China) is becoming more and more perfect, and the quality of infectious disease reporting is improving.^[13]^ A large-scale quality control survey showed that the average underreporting rate of notifiable infectious diseases in China fell from 23.14% in 2005 to 4.35% in 2015.

In this study, we noticed that the peak trend of infectious diarrhea in China from 2004 to 2017 was the same over the years, showing an apparent seasonal bimodal distribution, with frequent occurrence in winter and summer. Seasonal characteristics of infectious diseases are valuable resources for inferring spatio-temporal transmission parameters, which help to understand better and predict the spread of diseases.^[14,15]^ However, the seasonality of infectious diseases may be affected by geographical differences. Consistent with the previous research results, Beijing has a peak incidence in summer, and the peak incidence in Guangdong Province is concentrated in autumn and winter. The peak of diarrhea in winter may be due to the dry and cold season, which is suitable for the growth and reproduction of the virus. The peak of diarrhea in summer may be due to the high temperature, which bacteria are easy to grow and reproduce in, which pollutes water and food.^[16,17]^ Therefore, health education on foodborne diseases among residents and catering units should be carried out, and supervision should be strengthened to avoid raw and semi-raw aquatic products and their cross contamination.^[18]^

OID presented a specific temporal and spatial distribution usually clustered spatially in different geographical locations. The results of regional distribution show that the top 3 regions with the highest incidence rate are Tianjin, Beijing, and Zhejiang province. On the 1 hand, Tianjin, Beijing and Zhejiang Province are typical areas with rapid economic development in China. The medical and health capacity and disease control systems in these areas are highly scientific and standardized, which can control the number of missed diagnoses and reporting of infectious diseases at a low level. On the other hand, with the economic development in these areas, the social population flow has always been in an active state, and the unstable living conditions and environment provide a sociological basis for the prevalence of OID.^[19]^ From another point of view, in China, the incidence rate in China’s remote border provinces such as Yunnan and Xinjiang is increasing, which may be due to the combination of various factors that can promote the development and spread of infectious diseases in these areas, such as the different customs of the multi-ethnic population, imperfect food and drinking water safety mechanisms, limited access to health services, and low efficiency of public health programs and infrastructure.

Our research results show that the incidence rate of males in all age groups is higher than that of females. It may be related to men’s social mobility induced greater exposure to OID risk factors, poor personal hygiene habits, and backward health care awareness.^[20]^ In addition, the proportion of men who smoke and drink alcohol and bear family economic and social pressure is higher than that of women, so there is a high possibility of sub-health, which will lead to the decline of body immunity and increase the chance of infection. From the perspective of occupational distribution, scattered children are the main population of OID, accounting for 49.80% of the total cases, followed by farmers. The possible factors are mainly attributed to these 2 groups’ backward health awareness and hygiene habits. In addition, consistent with previous studies, the incidence rate of children is significantly higher than that of adults, and the incidence rate is the highest in the 0 to 4 years old group.^[21]^ T The reasons for this result may be related to the immature immune system of children, the imperfect development of the gastrointestinal function, and the failure to establish good hygiene habits.^[22,23]^ Therefore, it is necessary to strengthen the publicity and education of food safety knowledge and improve health awareness for families of children. In addition, the health department should pay attention to implementing the rotavirus vaccination plan.

Therefore, it is necessary to strengthen the publicity and education of food safety knowledge and improve health awareness for families of children. In addition, the health department should pay attention to implementing the rotavirus vaccination plan. Non-pharmaceutical intervention reduces the OID reported incidence rate, which should be attributed to 3 reasons. First, the containment and closure policies have affected the flow of people, and the dining together situation has been significantly reduced, which improved the hygiene of diet and drinking water to a certain extent.^[24]^ Second, the popularization of hand hygiene measures may play an essential role in reducing the incidence of gastrointestinal diseases.^[25]^ Third, we cannot ignore that some patients with mild OID disease give up the plan to go to medical institutions for a precise diagnosis due to their concerns about COVID-19 infection. Although it is still unclear which factor plays a more active role, this study adds epidemiological evidence that the prevention and control of COVID-19 are accompanied by the containment of the spread of other diseases. This discovery might bring a beneficial effect on the formulation of OID prevention and control policies.

In addition, this study inevitably has certain limitations. First, the data on OID mainly come from China’s legally infectious diseases report. In some regions, the disease incidence rate may be underestimated. Second, the geographical information of the data is relatively lacking, and the present analysis is only conducted on a national scale. Third, this study failed to confirm the real impact of Non-pharmaceutical interventions on OID in more detail according to the dynamic adjustment of epidemic prevention and control policies.

In conclusion, the epidemiological characteristics of OID in China present as follow: the incidence rate continuously increasing, bimodal distribution in summer and winter, inconspicuous regional characteristics, a higher incidence in children aged 0 to 4, and differential gender susceptibility. However, the COVID-19 prevention and control policy significantly reduced the number of reported cases of OID in 2020. The discoveries might bring a beneficial effect on the formulation of OID prevention and control policies.

## Author contributions

**Data curation:** Yujie Ge.

**Formal analysis:** Yujie Ge.

**Methodology:** Kai Wang, Jun Liu.

**Project administration:** Yujie Ge.

**Supervision:** Kai Wang.

**Writing – original draft:** Yujie Ge.

**Writing – review & editing:** Lingzhong Xu.

## References

[1] Harikrishnan S Jeemon P Mini G. Global, regional, and national age-sex-specific mortality for 282 causes of death in 195 countries and territories, 1980-2017: a systematic analysis for the global burden of disease study 2017. Lancet. 2018;392:1736–88. 30496103 10.1016/S0140-6736(18)32203-7 PMC6227606

[2] Troeger C Blacker BF Khalil IA. Estimates of the global, regional, and national morbidity, mortality, and aetiologies of diarrhoea in 195 countries: a systematic analysis for the Global Burden of Disease Study 2016. Lancet Infect Dis. 2018;18:1211–28. 30243583 10.1016/S1473-3099(18)30362-1 PMC6202444

[3] Siciliano V Nista EC Rosà T. Clinical management of infectious diarrhea. Rev Recent Clin Trials. 2020;15:298–308. 32598272 10.2174/1574887115666200628144128

[4] Yang S Wu J Ding C. Epidemiological features of and changes in incidence of infectious diseases in China in the first decade after the SARS outbreak: an observational trend study. Lancet Infect Dis. 2017;17:716–25. 28412150 10.1016/S1473-3099(17)30227-X PMC7164789

[5] WHO. Coronavirus disease (COVID-19) pandemic. Available at: https://www.who.int/emergencies/diseases/novel-coronavirus-2019 [access date July 6, 2022].

[6] Cheng ZJ Zhan Z Xue M. Public health measures and the control of COVID-19 in China. Clin Rev Allergy Immunol. 2021;1:1–16. 10.1007/s12016-021-08900-2 PMC8449219 34536214

[7] Yan X Wang X Zhang X. The epidemic of sexually transmitted diseases under the influence of COVID-19 in China. Front Public Health. 2021;9:737817. 34976912 10.3389/fpubh.2021.737817 PMC8716580

[8] Hu C-y Tang Y-w Su Q-m. Public health measures during the COVID-19 pandemic reduce the spread of other respiratory infectious diseases. Front Public Health. 2021;9:771638. 34858936 10.3389/fpubh.2021.771638 PMC8631357

[9] Douglas A Sandmann FG Allen DJ. Impact of COVID-19 on national surveillance of norovirus in England and potential risk of increased disease activity in 2021. J Hosp Infect. 2021;112:124–6. 33716087 10.1016/j.jhin.2021.03.006

[10] Wang LP Han JY Zhou SX. The changing pattern of enteric pathogen infections in China during the COVID-19 pandemic: a nation-wide observational study. Lancet Reg Health West Pac. 2021;16:100268. 34568854 10.1016/j.lanwpc.2021.100268 PMC8450280

[11] Li W Li W Li L. Multiplex detection of eight different viral enteropathogens in clinical samples, combining RT-PCR technology with melting curve analysis. Virol J. 2022;19:61. 35392937 10.1186/s12985-022-01789-z PMC8991609

[12] Gao X Hao J Yu L. Evaluation of enterovirus nucleic acids detection method based on ultra-fast real-time fluorescence RT-PCR technology-A pilot study. J Med Virol. 2022;94:9. 10.1002/jmv.27886 35619216

[13] Jiang Y Dou X Yan C. Epidemiological characteristics and trends of notifiable infectious diseases in China from 1986 to 2016. J Glob Health. 2020;10:020803. 33214900 10.7189/jogh.10.020803 PMC7649044

[14] Han C Li M Haihambo N. Enlightenment on oscillatory properties of 23 class B notifiable infectious diseases in the mainland of China from 2004 to 2020. PLoS One. 2021;16:e0252803. 34106977 10.1371/journal.pone.0252803 PMC8189525

[15] Lutz CS Huynh MP Schroeder M. Applying infectious disease forecasting to public health: a path forward using influenza forecasting examples. BMC Public Health. 2019;19:1659. 31823751 10.1186/s12889-019-7966-8 PMC6902553

[16] Wang G Zhao RQ Tang X. Age-specific spectrum of etiological pathogens for viral diarrhea among children in twelve consecutive winter-spring seasons (2009–2021) in China. J Med Virol. 2022;94:3840–6. 35441419 10.1002/jmv.27790 PMC9324210

[17] Xu Z Hu W Zhang Y. Exploration of diarrhoea seasonality and its drivers in China. Sci Rep. 2015;5:8241. 25649629 10.1038/srep08241 PMC4316158

[18] Thiede H Duchin JS Hartfield K. Variability in practices for investigation, prevention, and control of communicable diseases among Washington State’s Local health jurisdictions. J Public Health Manag Pract. 2012;18:623–30. 23023289 10.1097/PHH.0b013e3182602f90

[19] Wu T Perrings C Kinzig A. Economic growth, urbanization, globalization, and the risks of emerging infectious diseases in China: a review. Ambio. 2017;46:18–29. 27492678 10.1007/s13280-016-0809-2 PMC5226902

[20] Covan EK. Infectious communicable diseases: are gender differences due to social structure or biology. Health Care Women Int. 2021;42:249–50. 33970829 10.1080/07399332.2021.1920812

[21] Chen C Guan Z Huang C. Epidemiological trends and hotspots of Other Infectious Diarrhea (OID) in Mainland China: a population-based surveillance study from 2004 to 2017. Front Public Health. 2021;9:679853. 34368054 10.3389/fpubh.2021.679853 PMC8339203

[22] Winskill P Hogan AB Thwing J. Health inequities and clustering of fever, acute respiratory infection, diarrhoea and wasting in children under five in low- and middle-income countries: a demographic and health surveys analysis. BMC Med. 2021;19:144. 34162389 10.1186/s12916-021-02018-0 PMC8223394

[23] Ye Z Huang Y Zheng C. Clinical and genetic spectrum of children with congenital diarrhea and enteropathy in China. Genet Med. 2019;21:2224–30. 30894704 10.1038/s41436-019-0488-z

[24] Lai S Ruktanonchai NW Zhou L. Effect of non-pharmaceutical interventions for containing the COVID-19 outbreak in China. medRxiv. Mar 6 2020; doi:10.1101/2020.03.03.20029843

[25] Lin R Lin S Yan N. Do prevention and control measures work? Evidence from the outbreak of COVID-19 in China. Cities. 2021;118:103347. 34312572 10.1016/j.cities.2021.103347 PMC8282487

